# Planning for the next influenza H1N1 season: a modelling study

**DOI:** 10.1186/1471-2334-10-301

**Published:** 2010-10-21

**Authors:** Fabrice Carrat, Camille Pelat, Daniel Levy-Bruhl, Isabelle Bonmarin, Nathanael Lapidus

**Affiliations:** 1 Université Pierre et Marie Curie - Paris 6, UMR-S 707, Paris, F-75012, France; 2 Inserm U707, Paris, F-75012, France; 3 Assistance Publique Hôpitaux de Paris, Hôpital Saint Antoine, Paris, F-75012, France; 4 Département des maladies infectieuses, Institut de Veille Sanitaire, Saint-Maurice; 94415, France; 5 F Carrat, UMR-S 707, Faculté de médecine Saint Antoine, 27 rue Chaligny, 75571 PARIS CEDEX 12, France

## Abstract

**Background:**

The level of herd immunity before and after the first 2009 pandemic season is not precisely known, and predicting the shape of the next pandemic H1N1 season is a difficult challenge.

**Methods:**

This was a modelling study based on data on medical visits for influenza-like illness collected by the French General Practitioner Sentinel network, as well as pandemic H1N1 vaccination coverage rates, and an individual-centred model devoted to influenza. We estimated infection attack rates during the first 2009 pandemic H1N1 season in France, and the rates of pre- and post-exposure immunity. We then simulated various scenarios in which a pandemic influenza H1N1 virus would be reintroduced into a population with varying levels of protective cross-immunity, and considered the impact of extending influenza vaccination.

**Results:**

During the first pandemic season in France, the proportion of infected persons was 18.1% overall, 38.3% among children, 14.8% among younger adults and 1.6% among the elderly. The rates of pre-exposure immunity required to fit data collected during the first pandemic season were 36% in younger adults and 85% in the elderly. We estimated that the rate of post-exposure immunity was 57.3% (95% Confidence Interval (95%CI) 49.6%-65.0%) overall, 44.6% (95%CI 35.5%-53.6%) in children, 53.8% (95%CI 44.5%-63.1%) in younger adults, and 87.4% (95%CI 82.0%-92.8%) in the elderly.

The shape of a second season would depend on the degree of persistent protective cross-immunity to descendants of the 2009 H1N1 viruses. A cross-protection rate of 70% would imply that only a small proportion of the population would be affected. With a cross-protection rate of 50%, the second season would have a disease burden similar to the first, while vaccination of 50% of the entire population, in addition to the population vaccinated during the first pandemic season, would halve this burden. With a cross-protection rate of 30%, the second season could be more substantial, and vaccination would not provide a significant benefit.

**Conclusions:**

These model-based findings should help to prepare for a second pandemic season, and highlight the need for studies of the different components of immune protection.

## Background

On 11 June 2009, WHO announced the first influenza pandemic of the 21st century, following the emergence of a new influenza A/H1N1 virus in Mexico and its rapid worldwide spread. By March 2010 most countries had experienced a season of pandemic influenza H1N1, with one or occasionally two peaks. Surveillance reports showed that the burden of illness during this first season did not differ much from that of recent seasonal influenza epidemics [1-4], apart from a risk of unusually severe pneumonia in young people[5-15]. However, the true infection rates in the general population remain poorly documented.

Two parameters are of critical importance for interpreting surveillance data collected during this first pandemic season: first, the proportion of the population that was susceptible to infection before the 2009 pandemic influenza A/H1N1 virus (hereafter referred to as 2009 H1N1) started to circulate; and second, the rate of asymptomatic or paucisymptomatic infection. Several studies suggest that a substantial proportion of the population, and particularly the elderly, had pre-existing cross-reactive antibodies against 2009 H1N1 [16-18], and that asymptomatic or paucisymptomatic infection was relatively frequent [18,19]. Consequently, the level of pre- or post-exposure immunity is difficult to estimate, hindering attempts to predict the shape of a subsequent pandemic H1N1 season.

Here we estimate the infection attack rates during the first 2009 pandemic H1N1 season in France and attempt to predict the shape of a second season of pandemic H1N1 by using an individual-centred model [20]. We estimated the first season infection attack rates in three age groups: children, adults under 65, and the elderly, based on different postulates for the proportion of asymptomatic or paucisymptomatic infection. We then fitted the model to these attack rates according to pre-exposure cross-immunity and vaccine uptake, in order to derive the size of the immune population after the first pandemic season. Finally, we envisaged various scenarios in which pandemic influenza H1N1 viruses escaping immunity (due to viral evolution and loss of immunity) would be reintroduced, and evaluated the likely impact of extending 2009 H1N1 influenza vaccination.

## Methods

### Estimates of the infected population from national surveillance data

We used data from the French General Practitioner (GP) Sentinel network [21]. The network is a continuous epidemiological surveillance system based on voluntary GPs and operating since 1984 in France. Sentinel GPs report cases of influenza-like illness (ILI), defined as abrupt-onset fever above 39°C accompanied by respiratory signs and symptoms and myalgia or stiffness. Weekly national ILI incidence was estimated from the average number of ILI cases reported by GPs participating in surveillance during a week, multiplied by the ratio of all French GPs to participating sentinel GPs [22]. Surveillance criteria and procedures were not modified during the first pandemic season.

**Figure 1:**
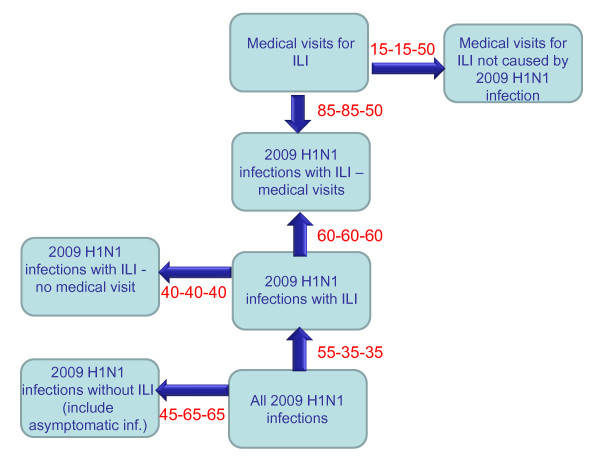
**Relationship between medical visits for influenza-like illness (ILI) and 2009 pandemic H1N1 infection**. Numbers associated with arrows indicate the percentages of the population from a source compartment that are expected in the next compartment, by age group (0-18, 19-64, ≥65)

Three age groups were considered: children (0-18 years), younger adults (< 65 years), and the elderly (≥65 years). In order to estimate the total size of the infected population, we took into account the fact that some cases of ILI might have been caused by other pathogens (poorly specific case definition), and that not all cases of influenza virus infection would result in ILI corresponding to the case definition (lack of sensitivity). The latter cases would include asymptomatic and paucisymptomatic infection. We also took into account the fact that not all patients with typical ILI seek medical advice (figure 1).

To overcome the poor specificity of the clinical case definition, we calculated the excess of GP consultations by children and adults under 65, relative to baseline rates, using seasonal regression models fitted to historical data since 1985, as described elsewhere [23]. The seasonal regression model was used to fit all-ages weekly incidence data between 1985 to 2010, defining the first French pandemic season as the period during which the incidence of medical visits for ILI exceeded the upper 90% limit of the predicted incidence for at least two consecutive weeks. The excess attributed to 2009 H1N1 was calculated in the same way, using a separate model for each age group, by summing the weekly differences between the observed and predicted incidence rates during the first pandemic season. We used a different method for elderly subjects, among whom the seasonality of medical visits for ILI was less clear-cut. We assumed that 50% of medical visits for ILI in this age group during the first pandemic season were associated with 2009 H1N1 infection, while rates of 0-100% were used to calculate confidence intervals. In children and younger adults, excess medical visits attributed to 2009 H1N1 represented, on average, 85% of all medical visits for ILI during the first pandemic season.

The proportion of patients with ILI who did not seek medical advice was estimated from a monthly telephone survey, conducted since May 2009, of a representative sample of 800 members of the general population (unpublished data). Overall, approximately 40% of persons who reported having typical 'flu-like symptoms did not consult a GP, which is consistent with the results of a prospective follow-up survey of 817 household contacts of index cases with seasonal influenza virus infection (43%) [24]. Finally, we used values from a meta-analysis of experimental human influenza challenge studies showing that approximately 65% of infected volunteers did not develop typical 'flu-like illness (33% did not have symptoms, 32% did not have fever), and who would not thus have matched our case definition [25]. The proportion of infections not matching the ILI definition was assumed to be lower in children (45%), who are considered more likely than adults to develop fever [26].

Population data and demographic parameters were obtained from national censuses [27]. In France, pandemic influenza vaccination started in November 2009, initially in groups at risk of complications. Influenza vaccine coverage rates were obtained from the French national security database. Adjuvanted vaccines were used in the vast majority of cases. At the end of first pandemic season the vaccine coverage rates were 12.6% in children, 6.8% in younger adults, and 6.7% in the elderly, most subjects having received a single injection. Confidence limits for proportions were calculated with the delta method.

### Fitting the first pandemic H1N1 season

We used an individual-centred model, which permits rich parameterization of the simulated population [20]. The model included detailed descriptions of healthcare use and interventions aiming at controlling influenza. It also included demographic characteristics and household sizes, and simulated the spread of influenza through the use of randomly generated graphs. The random graphs were a mixture of bidirectional graphs, comprising fully-connected graphs for describing contact pattern within the household, and Barabasi-Albert scale free graphs [28] for describing other social contacts. The networks exhibit a substantial level of clustering meaning that two simulated individuals have an increased chance to be contacts of each other given that they share a common network contact. The connectivity of the simulated network followed a power law distribution, with some individuals having a large number of contacts which allows generation of superspreading events

The mean number of contacts per subject (the connectivity of the network) was 13.9 overall (standard deviation SD = 0.4), 15.3 (SD = 0.06) for children, 14.6 (SD = 0.59) for younger adults, and 5.1 (SD = 0.16) for the elderly, in keeping with the results of recent large surveys [29,30]. New networks were generated at each simulation.

We made the following assumptions:

- We used realistic modelling of infectivity based on experimental infection viral shedding data [25]. Rather than assuming that infectivity was constant, we modelled it as a function depending on the time elapsed from infection [31]. We assumed the kinetics of infectivity had a gamma density function form (shape parameter = 5.2, scale parameter = 1), with an offset of 0.5 day (a latent period) and the function was truncated at ten days. Infectivity did not depend on age [32] and peaked at 2.1 days, with a calculated generation time of 2.6 days [33-35]. Infectivity was scaled during the fitting process to adjust the observed data. The resulting probability of transmission during a hypothetical meeting lasting throughout the infective period between a susceptible and a single infected individual was 40%.

- Children were fully susceptible to infection, and unknown proportions of the younger adult and elderly populations (to be calculated) were immune to 2009 H1N1 before it started to circulate. We assumed that these immune subjects could not be infected, irrespective of the number of contacts with infectious persons ("all-or-nothing" protection) [36].

- Subjects with asymptomatic infection were half as infective as other subjects, and, among subjects who consulted a GP, 40% did so the first day after symptom onset, 30% the second day, and 30% later than the second day [24].

- We postulated that 70% of individuals who consulted a GP would remain confined to home for five days, as recommended [37].

- We assumed that 50% of patients who visited a doctor within two days of symptom onset received antiviral therapy. We also assumed that antiviral treatment reduced an individual's infectiousness by 28% [38], and their risk of severe influenza by 80% [5,7,12,13]. Antiviral prophylaxis was not considered, as it was not recommended in France during the first H1N1 season.

- For consistency with observed vaccine coverage rates, we assumed that vaccination started 4 weeks after the outset of the epidemic and increased linearly over the next 7 weeks. We assumed that influenza vaccination was 80% protective against infection and illness, irrespective of age, starting 15 days after vaccination. Vaccination was administered irrespective of the individual's history of exposure or immunity to influenza viruses.

The model was calibrated by varying the proportion of younger adults and elderly subjects with pre-exposure immunity to fit the excess rates of medical visits attributed to 2009 H1N1 in the relevant age group. For each set of parameters we ran 400 simulations, starting with a single infectious individual at the first day of the first French pandemic season. We classified as "outbreaks" situations in which more than 5 per 1000 subjects were infected [20]. Goodness-of-fit was optimized by minimising the difference by age group between the observed and average rates in simulated outbreaks. The size of the post-exposure immunized population was estimated in each of the three age groups as the proportion of individuals who were infected during the first pandemic season or who were immunized naturally or by vaccination prior to the first pandemic season. Confidence limits for the rates of post-exposure immunity were calculated, assuming that the rate of pre-exposure immunity could vary between +5% and -5% of the values obtained in the fitted model.

We also simulated a scenario in which the entire population was susceptible before introducing infectious individuals, all other parameters being equal, in order to examine how a total lack of pre-exposure immunity might influence the first pandemic season.

We calculated the effective reproductive number by simulating the first generation of secondary cases after introducing a single infectious subject, as described elsewhere [20]. We also calculated a basic reproductive number by setting all parameters related to healthcare use (treatment, isolation, etc.), and the size of the pre-exposure immune population, to zero.

### Scenarios: reintroduction of pandemic H1N1 viruses with modified antigenic properties, varying levels of cross-protection, and different vaccination strategies

We simulated reintroduction of individuals infected by a pandemic H1N1 virus exhibiting modified antigenic properties (due to antigenic drift for example), at a rate of 2/1000, in a population in which individuals who were immune after the first season had varying levels of persistent protective cross-immunity against the new virus [39]. For these individuals, the chance of being infected during contact with an infected individual was reduced by 90% to 30%. All other parameters (transmission parameters, pathogenicity, contact networks and healthcare use) remained identical to those used to adjust the first epidemic curve. We completed each scenario by postulating that 10% to 50% of the entire population (or children) who were not vaccinated during the first pandemic season would receive the 2009 H1N1 vaccine before the reintroduction of infectious individuals. Vaccine effectiveness was reduced in proportion to the postulated cross-protection (vaccine effectiveness = 80% x cross-protection), as the mechanism underlying the loss of naturally acquired immunity would also concern vaccination-induced immunity, as vaccines being prepared for the 2010-2011 season cover 2009 H1N1 [40].

## Results

### Estimates of the infected population from national surveillance data

The first pandemic season in France lasted 16 weeks, from 7 September 2009 to 27 December 2009. The incidence of medical visits for ILI increased moderately and remained at a stable low level during the first 6 weeks, then increased more sharply and peaked between 6 and 12 December.

**Figure 2:**
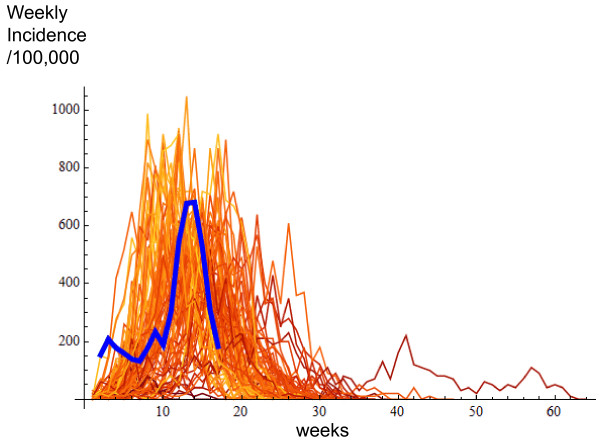
**Estimated excess medical visits for 2009 pandemic H1N1 infection during the first season (flat blue lines) and simulated curves of the first pandemic season obtained with the calibrated model in the pre-exposure immunized population (thin orange lines)**.

During the course of the first pandemic season, we estimated that the proportions of the population who consulted a GP for ILI were 4.86% overall (95%CI 3.62%-6.11%), 12.7% among children (95%CI 11.3%-14.0%), 3.11% (95%CI 1.67%-4.56%) among younger adults, and 0.34% among the elderly (95%CI 0-0.68%) (figure 2). The estimated proportion of the population infected by pandemic H1N1 was 18.1% overall (95%CI 12.2%-23.9%), 38.3% (95%CI 30.8%-45.9%) among children, 14.8% (95%CI 7.01%-22.6%) among younger adults, and 1.62% (95% CI 0%-3.60%) among the elderly.

### Fitting the first pandemic H1N1 season

The model was fitted to the excess rates of medical visits attributed to 2009 H1N1 by setting the pre-exposure immune populations to 36% among younger adults and 85% among the elderly. The simulated proportion of infected persons was 18.2% overall (InterQuartile Range (IQR) 17.2%-20.7%), 39.3% in children (IQR 37.3%-44.0%), 14.8% in younger adults (IQR 13.0%-17.1%) and 1.49% in the elderly (IQR 1.21%-1.76%). The simulated outbreaks lasted an average of 13.1 weeks (IQR 11-14 weeks), 10% of outbreaks exceeding 16 weeks.

The post-exposure immune population represented 57.3% overall (95%CI 49.6%-65.0%), 44.6% (95% CI 35.5%-53.6%) in children, 53.8% (95%CI 44.5%-63.1%) in younger adults, 87.4% (82.0%-92.8%) in the elderly.

Postulating no pre-exposure immunity in younger adults and elderly persons, the simulated proportions of infected persons would be 47.9% overall (IQR 46.2%-49.7%), 64.2% in children (IQR 62.6%-65.4%), 47.6% in younger adults (IQR 45.4%-49.7%) and 26.7% among the elderly (IQR 25.2%-27.9%). An estimated 11.9% (11.4%-12.4%) of the total population would consult a GP for ILI caused by 2009 H1N1.

**Figure 3:**
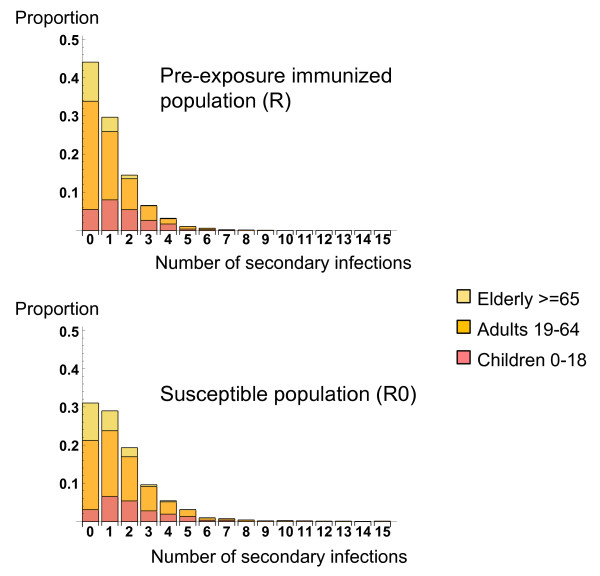
**Distribution of the number of secondary infections engendered by a single infectious individual in a population of younger adults and elderly subjects with pre-exposure immunity (R) and in an entirely susceptible population (R0), according to age group.** 4000 simulations were made.

The effective reproductive number was 1.03 and the basic reproductive number 1.54 (figure 3). An average of 1.57 persons were infected (1.11 children, 0.45 younger adults and 0.01 elderly persons) when the index patient was a child, 0.96 (0.23 children, 0.72 younger adults, 0.01 elderly) when the index patient was a younger adult, and 0.43 (0.14 children, 0.25 younger adults, 0.04 elderly persons) when the index patient was an elderly person.

### Scenarios: reintroduction of pandemic H1N1 viruses with modified antigenic properties, varying levels of cross-protection, and different vaccination strategies

As shown in Table 1, persistent cross-protection above 70% would be necessary to markedly limit the size of a second pandemic season due to a virus with different antigenic properties. Cross-protection of 50% would imply attack rates of 23.1% overall, and rates notably higher in younger adults and the elderly than during the first pandemic season. Influenza vaccination of 30 to 50% of the population, in addition to the population vaccinated during the first pandemic season, would halve the attack rates, provided that cross-protection was not below 50%. With cross-protection below 30%, vaccination of 50% of the population would have little impact. Vaccination of children alone would limit the burden of influenza in all age groups if cross-protection was between 50% and 70% and the vaccine coverage rate exceeded 50%; in contrast, this measure would have little impact in case of cross-protection greater than 70%; and would be barely effective in case of cross-protection below 50%.

**Table 1:** Simulated infection rates during a second pandemic influenza H1N1 season according to persistent cross-protection and increasing vaccine coverage

		Increased vaccine coverage (%)					
		**All ages**			**0-18 yrs**		

**Persistent protective cross-immunity (%)**	**No vaccination**	**10**	**30**	**50**	**30**	**50**	**70**

90	2.35 (0.66-2.85) 1.76 (0.48-2.49) 0.29 (0.12-0.36) **1.65 (0.51-2.31)**	1.42 (0.53-1.89) 1.11 (0.31-1.35) 0.16 (0.06-0.18) **1.02 (0.35-1.35)**	0.68 (0.26-0.88) 0.59 (0.20-0.72) 0.10 (0.00-0.12) **0.53 (0.23-0.66)**	0.30 (0.13-0.39) 0.26 (0.12-0.30) 0.05 (0.00-0.06) **0.23 (0.13-0.26)**	1.05 (0.39-1.58) 1.40 (0.33-1.86) 0.21 (0.06-0.30) **1.12 (0.34-1.44)**	0.65 (0.26-0.88) 1.21 (0.33-1.70) 0.14 (0.00-0.18) **0.91 (0.27-1.21)**	0.40 (0.13-0.57) 1.26 (0.31-1.94) 0.16 (0.06-0.24) **0.88 (0.24-1.32)**

70	14.0 (11.2-18.4) 10.3 (7.33-14.3) 2.65 (1.94-3.63) **9.91 (7.29-13.9)**	8.44 (2.68-13.8) 6.17 (1.66-10.4) 1.56 (0.55-2.48) **5.92 (1.71-9.82)**	3.51 (0.83-5.04) 2.81 (0.59-3.94) 0.66 (0.18-0.97) **2.61 (0.59-3.65)**	1.24 (0.48-1.62) 1.13 (0.30-1.20) 0.29 (0.06-0.36) **1.02 (0.32-1.18)**	6.20 (1.54-9.96) 6.05 (1.63-10.2) 1.45 (0.42-2.42) **5.32 (1.37-8.78)**	4.76 (1.36-7.11) 6.56 (1.45-10.2) 1.46 (0.42-2.18) **5.31 (1.31-8.39)**	2.73 (0.66-4.30) 4.61 (0.69-8.14) 1.06 (0.18-1.76) **3.60 (0.63-6.20)**

50	33.2 (30.9-36.3) 23.1 (20.7-25.8) 8.85 (7.70-10.1) **23.1 (20.7-25.2)**	29.4 (26.3-33.3) 21.3 (18.4-24.3) 7.99 (7.09-9.03) **20.9 (18.6-23.5)**	20.2 (18.1-25.4) 15.5 (12.0-20.4) 5.76 (4.73-7.27) **15.0 (12.4-19.2)**	14.0 (9.91-19.4) 11.6 (6.95-15.5) 4.16 (2.55-5.82) **10.9 (0.69-15.2)**	25.6 (23.8-28.4) 20.9 (18.4-24.2) 7.82 (6.91-8.97) **19.8 (18.0-22.8)**	21.4 (19.6-24.6) 19.5 (16.4-22.8) 7.13 (6.42-8.18) **17.9 (15.6-20.6)**	17.3 (14.9-21.8) 18.7 (16.0-23.7) 6.47 (5.33-8.24) **16.4 (13.7-20.7)**

30	49.7 (48.3-51.4) 35.2 (32.5-37.8) 16.6 (15.2-17.9) **35.4 (33.2-37.5)**	46.5 (44.9-48.9) 34.0 (31.6-36.3) 15.7 (14.4-17.0) **33.8 (32.3-36.2)**	43.6 (41.3-45.9) 31.7 (28.9-33.7) 14.7 (13.6-15.8) **31.6 (29.1-33.4)**	39.7 (37.5-42.5) 29.1 (27.2-31.6) 13.7 (12.8-14.5) **29.0 (27.3-31.1)**	45.1 (43.0-47.1) 34.6 (32.4-36.9) 15.9 (14.9-16.9) **33.9 (31.9-35.8)**	41.7 (40.2-44.0) 32.6 (30.3-35.3) 15.1 (13.9-16.6) **31.8 (30.2-34.0)**	39.8 (37.7-42.4) 32.8 (30.0-36.0) 15.2 (14.1-16.4) **31.5 (29.2-34.2)**

In each cell, mean infection rates per 100, in children (0-18 years), adults under 65 years and elderly (standard style) and total population (bold style) are given with their interquartile ranges (in parentheses).

## Discussion

Between 12% and 24% of the French population were infected by the 2009 H1N1 pandemic virus during autumn and winter 2009. The cumulative incidence rate was much higher in children (38.3%) than in younger adults (14.8%) and the elderly (1.62%).

Here, using a modelling approach parameterized with the best available epidemiological data for the French 2009 H1N1 season, and taking into account the behaviour of patients with ILI and implementation of control measures, we show that substantial pre-exposure immunity to 2009 H1N1 -- 36% in younger adults and 85% in the elderly -- would be necessary to fit the observed incidence rates.

The likelihood of a major second 2009 H1N1 season would depend on the degree of persistent protective cross-immunity against new 2009 H1N1 variants. Assuming cross-protection of 70% among people infected during the first 2009 H1N1 season and among those who were already protected, a second season would affect only a small proportion of the population. With cross-protection of 50%, the second season would have a disease burden similar to that of the first season, and vaccination of 50% of the entire population, in addition to the population vaccinated during the first pandemic season, would more than halve this burden. With cross-protection of 30%, the second season could be substantial, even if vaccine coverage increased by 50%.

Our estimates of the cumulative incidence rates of 2009 H1N1 infection during the first pandemic season are slightly higher than those obtained in a serological survey conducted in London and the West Midlands, which suggested that 21.3% of < 5-year-olds, 42.0% of 5- to 14-year-olds, 20.6% of 15- to 24-year-olds, 6.2% of 25- to 44-year-olds and only 0.9% of the elderly were infected [18]. These incidence rates were based on differences in the proportion of samples with haemagglutination inhibition titres of 1:32 or higher between 2008 and September 2009, and did not take into account 2009 H1N1 infections occurring in late October-early November 2009. Also, 11% of individuals who had PCR-confirmed 2009 H1N1 infection had not seroconverted after 21 days, indicating that 2009 infection rates were underestimated. In another study, post-exposure 2009 H1N1 seroprevalence rates were 45% in children aged 10 to 19 years, 14% to 22% in adults under 60, and 5% to 26% in adults aged 60 to 89 [41], giving an overall rate of 21.5%. Our findings are compatible with these data. Interestingly, in this latter study, several findings pointed to protective cross-immunity due to exposure to previous H1N1 viruses. In the elderly population, antibodies to the 1918 H1N1 virus were found in 48% to 57% of cases, while antibodies to the 1957 H1N1 virus (a descendant of 1918 H1N1) were found in 37% to 58% of adults aged 40 to 59. Cross-reactivity between antibodies elicited by H1N1 viruses circulating up to 1957 is further supported by the antigenic similarity [42] and reported cross-neutralization [43] between these viruses and 2009 H1N1. Together, these findings help to explain the low attack rates observed in the adult and elderly populations. However, the precise level of pre- or post-exposure immunity to 2009 H1N1 is difficult to evaluate. Serological analyses only measure adaptive immunity, failing to quantify cellular and innate immunity. Moreover, the protection associated with haemagglutination inhibition or neutralization antibody titres is not known for 2009 H1N1.

We found evidence of elevated levels of pre-exposure immunity in the French adult and elderly populations, yielding a reproductive number slightly higher than 1. Such a low reproductive number is supported by the highly variable patterns of the first 2009 H1N1 seasons across various countries, with one or two peaks, a lack of spatial synchrony, and moderate clinical attack rates [44]. We estimated the reproductive number on our data using the early epidemic growth rate (fitted on incidence data from one week before to two weeks after the epidemic onset, using the method described in [45], with a generation time of 2.6 days and a ratio of infectious period to the generation time of 0.61). The value obtained (1.18) was close to our estimate and lower than the average estimate of 1.3 during seasonal influenza epidemics in France [46]. Estimates of the final size of the infected population, taking into account relative susceptibilities in different age groups also closely matched our estimates (17.5%) [47]. However, our reproductive number was substantially lower than those reported in the US or Mexico for the first pandemic season [33,34]. This apparent discrepancy may be the consequence of estimations in different time, settings or countries but may also be explained by our modelling framework. In scale-free networks of finite size, the heterogeneity of scale-free connectivity patterns favors epidemic spreading by lowering the epidemic threshold [48]. In contrast with homogeneous network, the scale-free network allows epidemic spreading for a low average number of infections produced by an infected individual [49].

Our calculations of pre-exposure protective cross-immunity may have been influenced by assumptions concerning the proportion of infected individuals who did not develop ILI. It has been estimated that the proportion of all infected subjects who visited their GP in France was 19.6% among pregnant women [19], a figure in line with our postulate of 21% in the 19- to 64-year age group. When we postulated lower proportions of individuals with ILI among those infected with 2009 H1N1 [18], the cumulative incidence rates of infection increased and the level of pre-exposure immunity necessary to fit the epidemic curve therefore decreased. In this case, transmission parameters would also increase, and our estimates of the post-exposure immunized population would not be markedly affected.

There are few reports on cross-protection between successive pandemic or seasonal influenza seasons. The cross-protective effect was estimated to range from 35% to 94% for clinical illness between the spring and summer waves and the autumn wave during the 1918 pandemic [50], while no evidence of cross-protection was found between the autumn wave and a third winter wave. As the 1918 pandemic H1N1 virus was antigenically close to 2009 H1N1, a strong decline in protection could occur if the same situation is repeated. Genetic characterization of 2009 H1N1 has already identified different evolving clades and complex spatio-temporal dynamics [51], and significant drift before the next season is likely.

Mass vaccination with the 2009 H1N1 influenza vaccine would be effective only within a limited range of cross-protection against a re-emerging H1N1 strain. Even if cross-reactive antibodies might have been elicited by adjuvanted influenza vaccination (used in late 2009/early 2010 in France), there is no evidence that adjuvanted vaccines provide superior cross-protection than naturally-acquired infection against drifted strains, and we therefore applied the same reasoning to individuals who were naturally immunized and those who were vaccinated.

The next pandemic season, if it occurs, could affect more adults and elderly subjects than the first. This was the case in past pandemics [52] and has been carefully analyzed in a network-based modelling study, in which a shift to older age between the first and subsequent seasons was predicted [53]. As the case-fatality ratio was higher in the adult and elderly populations than in children [15,54], the mortality burden of a subsequent H1N1 pandemic season due to a virus with unchanged pathogenicity could be higher than during the first season.

## Conclusions

Pre-exposure immunity to 2009 H1N1 influenza virus was higher than anticipated in French adults and elderly people. A sustained high level of cross-protection against descendants of the 2009 H1N1 virus would be necessary to avoid a second significant 2009 H1N1 season. Extending influenza vaccination across all age groups would be effective if cross-protection against descendants of the 2009 H1N1 virus ranged between 30% to 70%, but would not provide a significant benefit in other situations. This study therefore highlights the need for comprehensive studies of the different components of immune protection, and the need to maintain worldwide virological and ILI surveillance for early detection of antigenic drift.

## Abbreviations

WHO: World Health Organization; ILI: Influenza-Like Illness; GP: General Practitioner; SD: Standard Deviation; 95%CI: 95% Confidence Interval; IQR: InterQuartile Range.

## Competing interests

FC was a consultant for Novartis and GlaxoSmithKline; CP, DL-B, IB, NL have no relationships with companies that might have an interest in the submitted work in the previous 3 years; their spouses, partners, or children have no financial relationships that may be relevant to the submitted work; and (4) FC, CP, DL-B, IB, NL have no non-financial interests that may be relevant to the submitted work

## Authors' contributions

FC conceived the study and designed it with DLB. CP and NL collected data and did experiments for the study. FC, CP, DLB, and NL analyzed the data. FC wrote the first draft of the paper. FC, CP, DLB, IB and NL revised the paper. All authors read and approved the final manuscript.

## Pre-publication history

The pre-publication history for this paper can be accessed here:

http://www.biomedcentral.com/1471-2334/10/301/prepub
